# Commercial laying hens exhibit long-term consistent individual differences and behavioural syndromes in spatial traits

**DOI:** 10.1098/rsos.230043

**Published:** 2023-05-24

**Authors:** Camille M. Montalcini, Matthew B. Petelle, Michael J. Toscano

**Affiliations:** ^1^ ZTHZ, Division of Animal Welfare, VPH Institute, University of Bern, 3052 Zollikofen, Switzerland; ^2^ Graduate School of Cellular and Biomedical Sciences, University of Bern, 3012 Bern, Switzerland

**Keywords:** inter-individual variability, personality, farm animal, repeatability, movement, space-use

## Abstract

Past research has supported the importance of animal personalities for the productivity and welfare of farm animals. However, current assessments of personality traits are commonly conducted over short periods using standardized assays and may not reflect all important aspects of behaviours in commercial settings throughout the production period. This study aimed to evaluate consistent behavioural differences between 194 commercial laying hens within an aviary across most of the production period (eight months). We used five spatial behaviours related to various aspects of commercial hens' daily routine, including the sleeping, feeding, nesting, indoor movements and outdoor usage. All behaviours were repeatable over time and across contexts, with consistent differences between individuals explaining between 23% and 66% of the variation. These long-term consistencies revealed the potential applicability of the behaviours as personality traits of commercial hens. Moreover, we identified behavioural syndromes comprising all behaviours except the nesting-related behaviour, indicating two axes of spatial personalities that may be driven by different mechanisms. We discussed the significance of such individual differences in using personality traits to breed more resilient farm animals. Future research should evaluate associations of these behaviours with animal welfare and productivity to inform breeding efforts.

## 1. Introduction

Animal personality is defined as repeatable individual differences in behaviour over time and across contexts [1,2]. Personality traits can limit behavioural plasticity and hinder individuals from behaving optimally in all situations. That is, if a behaviour is not plastic enough, it will be suboptimal in some contexts. For example, high levels of feeding activity will be optimal when predator abundance is low but suboptimal when predator abundance is high due to higher predation risk. Therefore, studying correlations of a behaviour across contexts could explain suboptimal behaviours and identify important trade-offs driving individuals’ fitness [3]. Personality is a multi-dimensional concept and can be summarized into five axes: aggressiveness, activity, exploration, boldness and sociability [1,4]. These axes are often correlated into a behavioural syndrome [5]. More aggressive individuals tend to be more active, explorative, bold, as well as less social, and would commonly be classified as proactive animals (in contrast to reactive animals) [4,6,7]. Correlated traits can constrain evolution and help to understand key ecological processes and life-history strategies, such as population dynamics and survival (e.g. ‘fast’/proactive personalities [8] tend to disperse over longer distance [9] and activity—risk-taking syndrome affecting survival [10]). Importantly, behavioural traits can also be correlated to morphological and physiological traits, which is of particular importance in farm animals.

Indeed, there is a growing interest in farm animal personalities owing to their association with individual welfare and productivity. For instance, previous literature suggested that less nervous cows produced more milk [11], more exploratory-active calves associated with greater average daily gain [12], and calmer temperament in cows were related with greater first lactation milk yield [13]. A greater understanding of farm animal personality could help to improve management practices and the design of housing systems for increased productivity and welfare. Research suggested another critical role of animal personalities for the welfare of farm animals by proposing the integration of personality traits as phenotypes into the breeding process to breed for more robust farm animals [4,14,15] (e.g. in pigs [16], laying hens [17], cows [18]). Yet, personality traits are commonly assessed in laboratory settings or with standardized assays [19] on a limited number of individuals and over short periods of time. As a result, these traits may not reflect all important aspects of behaviours expressed within commercially relevant settings [20,21], which could lead to important misinterpretations that could impact how animals are housed, managed and bred. Therefore, technologies that allow automatic monitoring within commercial settings will be key in assessing farm animal personalities.

Following the incorporation of monitoring technology in the study of animal behaviour, how animals use their space is garnering increased attention [22]. Movement and space-use are fundamental behaviours for common personality traits [9] such as boldness, activity or exploration behaviour. Spatial behaviours are of particular importance in farm settings where animals have to live within complex human-made housings and where the high animal density and bounded environment constrain freedom of movement. The importance of spatial behaviours is especially true for commercial laying hens, where cage-free housing systems are becoming more common. Although thought to provide improved welfare over cage housing, cage-free housing is associated with certain welfare issues (e.g. severe feather pecking, bacterial infections and keel bone fractures [23]) that are more difficult to resolve with traditional management and genetic interventions. We suggest that by using tracking technologies that allow high-resolution, individual-level observations over long periods of time, we can establish new spatial behavioural traits in laying hens that can in turn be interpreted as personality traits, give insights into animal needs and preferences, and offer a promising tool to breed more resilient farm animals.

This study aimed to evaluate consistent behavioural variation between commercial laying hens within an aviary system across most of the production period. Our objectives were to: (i) characterize five spatial behaviours related to a hen's daily routine including: sleeping, feeding, nesting, indoor movements and outdoor usage, (ii) quantify the extent of consistent inter-individual differences (repeatability) over time and within four contexts for each behaviour, (iii) evaluate the maintenance of these individual differences across contexts, and (iv) explore the existence of behavioural syndromes. We hypothesized that such behaviours are indicative of personality traits and therefore predicted that all five behaviours would be repeatable over time and across contexts. We further hypothesized that the underlying personality traits are indicative of proactive and reactive strategies, and therefore predicted the behaviours to be correlated into behavioural syndromes.

## 2. Material and methods

### 2.1. Study design

The study was conducted according to the cantonal and federal regulations for the ethical treatment of experimentally used animals and approved by the Bern Cantonal Veterinary Office (BE-45/20). As part of a larger study, 2520 chicks (*Gallus gallus domesticus*) were reared in one of four rearing pens (630 chicks/pen), where half of the chicks hatched on farm (OFH treatment) while the other half arrived at 1 day of age from a commercial hatchery (TRAN treatment). Chicks originated from a single parent flock from a standard commercial hybrid (DeKalb White). At 7 days of age (DOA), all chicks were classified into a more/less explorer class (MEXP/LEXP). We will not use the class as an exploratory behaviour as the measurement could not be validated (electronic supplementary material, text S1), but we will control for the class in subsequent analysis. At 7 DOA, we selected 160 focal chicks (40/rearing pen; arbitrary selection of 10 animals among each class and 20 animals among the entire population). On the same day, we arbitrarily assigned (but not yet transferred) each focal chick to one of four identical laying pens from the same treatment (four pens/treatment for a total of eight laying pens, with 20 focal hens each), maintaining an equal representation of an individual's class and rearing pen throughout the laying pens. At 17 weeks of age (WOA; September 2020), we transferred all hens, including focals, to an onsite laying barn containing a Bolegg Terrace aviary (separated into 20 pens by grids; pens' indoor area: 7 m length, 2.3 m width, 2.69 m height until the top tier grid floor) and an outside covered winter garden (WG). Animal density was 8.1 hens m^−2^ of permanent accessible area (225 hens/pen of 27.92 m^2^). At five timepoints (127, 173, 243, 313, 417 DOA), we randomly selected 16 focal hens (two hens/pen) to be killed as part of the larger study and replaced them with 16 arbitrarily selected hens to continuously track the same number of hens (see below for descriptive statistics that accounts for technical issues). At five slightly different timepoints (173, 215, 243, 313, 417 DOA), all focal hens were weighed and radiographed to produce a latero-lateral image that was then used to generate a keel bone fracture (KBF) severity (continuous, 0–100) based on a tagged visual analogue scale [24]. The KBF score is an indicator of the total amount of the keel bone affected by any fracture (intra-observer reliability: intraclass correlation coefficient (ICC) = 0.89, 95% CI = 0.74–0.95, inter-observer reliability: ICC = 0.92, 95% CI = 0.832–0.96). Both rearing and laying barns are located at the Aviforum facilities in Switzerland where standard animal husbandry practices are used. Hens were kept for commercial and experimental purposes until July 2021.

### 2.2. Tracking system

**Figure 1.**
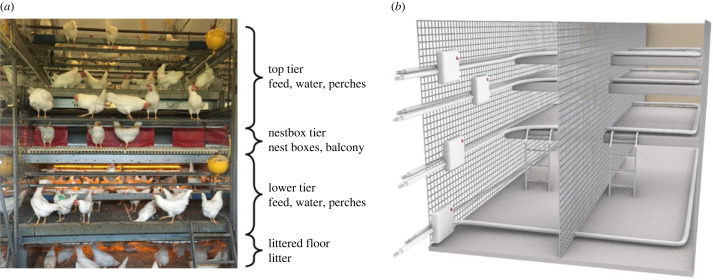
(*a*) Side view of the aviary including a single pen with its three aviary tiers (top tier, nestbox tier, lower tier) and the littered floor. (*b*) Simplified representation of the tracking system covering two pens, including the four indoor markers (square boxes) and their cables through which the signals are emitted (image adapted from [25]).

We tracked focal hens across five zones in the laying pen: the top and lower tiers (both containing water, feed and perches), the nestbox tier (with a balcony and nestboxes), the littered floor and the outside WG (containing water and litter). The four indoor zones are illustrated in figure 1*a*. We used a tracking system with active tags (mass: 28.1 g) enclosed in a backpack worn by each focal hen since the day of transfer to the laying barn (i.e. 17 weeks of age). The tracking system is composed of one marker per zone that emits low frequency (0.125 MHz) signals (every 1.6–2.1 s depending on the marker) through a cable creating separate enclosed fields for each zone (illustrated by figure 1*b*). However, at most locations in the laying pen, the tag can receive the signal from multiple zones. Therefore, every time a tag receives a signal, an algorithm is applied on the strength of all signals received by the tag within the past 10 s to determine if the hen has transitioned to a new zone (see Montalcini *et al*. [25] for detailed description and validation). We used the movement data from the point at which the daily number of eggs laid by the flock peaked and the management schedule (i.e. timing of lighting and winter garden access) became stable (i.e. at 25 weeks of age), until the end of production (i.e. at 60 weeks of age). During the tracking period, artificial light was turned on at 02.00 and off at 17.00. The dataset included 194 hens tracked over 242 days (i.e. eight months) resulting in a total of 30 780 observations (after removal of non-functional tags or days), among which hens had on average 159 days tracked (min = 12, max = 200, s.d. = 52, 25th percentile = 120, 50th percentile = 193, 75th percentile = 198 (unit: day)).

### 2.3. Spatial behaviours

**Figure 2.**
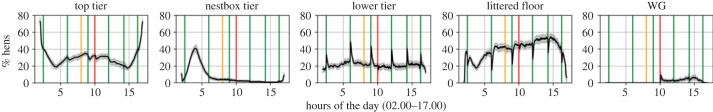
Black lines correspond to the mean (± s.d.) percentage of focal hens in a zone at specific time of the day, computed over all available days in January (arbitrarily chosen). Focal hens represent approximately 9% of the hens in each of the eight pens. The main daily husbandry practices are highlighted in coloured vertical lines (orange: collecting eggs on the floor; red: access to WG opened; green: fresh feed delivery).

We selected five daily spatial behaviours based on five aspects of commercial hens’ daily routine, so that behaviours may be functionally different. We attempted to avoid behaviours that would be intrinsically correlated, so that any behavioural syndromes that we identified should arise from individual hens' preferences. We hypothesized that hens with a more proactive personality would: travel greater vertical distances, use the WG on more days, have earlier nesting behaviours, spend more nights on the highest tier and express a stronger reaction to the feed being delivered by using the tier with feed more when fresh feed is available. Therefore, we predicted positive among-individual correlations (i.e. behavioural syndromes) between these behaviours. Figure 2 illustrates the percentage of focal hens in a zone at specific time of the day, along with the timing of the main husbandry practices.

#### 2.3.1. Vertical travelled distance

Because the four indoor zones are stacked on top of each other, we used the number of indoor zones crossed as a representation of a hen daily vertical travelled distance. More specifically, we used the mean vertical travelled distance per hour excluding the dusk, night and dawn phases and the time when the hen was in the WG to prevent overlap with the sleeping and outdoor usage behaviours. We tracked movements between zones but not within them, and thus did not measure hens’ activity. However, we hypothesized that hens that travel greater vertical distances would on average be more active and thus have a more proactive personality.

#### 2.3.2. Winter garden presence

The outside covered WG (9.32 m^2^) could be accessed via the littered floor through a pop-hole (length: 60 cm, height: 38 cm, width: 28 cm) from 10.00 until approximately 16.00 on most days. Most of its area cannot be seen from the inside and individuals must go to the edge of the pop-hole to see the entire WG. Although the WG is screened, it is subjected to more variable environmental conditions than the indoor area, which may cause some animals to perceive it with greater uncertainty. Previous research in free-range housing found negative association between range-use and fearfulness [26–28], although free-range area differs from our WG (i.e. no uncovered areas or access to grass). We used whether the hen entered the WG on the day (scored 0–no/1–yes) as the daily outdoor usage behaviour. We hypothesized that hens using the WG on more days would be less fearful and thus would have a more proactive personality.

#### 2.3.3. Nestbox tier timing

Nesting behaviour is a highly ritualized and internally motivated process [29,30]. In cage-free systems, hens should lay their eggs in shared nestboxes that cannot hold all hens simultaneously (here: 2.3 m^2^ of nestbox surface per pen of 225 hens). Therefore, dominance hierarchies may affect laying behaviour, with subordinate hens laying their eggs (and potentially also performing nesting behaviour) slightly later than dominant hens [31]. Therefore, to account for nesting behaviour, we used the period when hens are expected to lay (i.e. 02.00–08.00) and extracted the point in time (h) when a hen reached half of its time spent in the nestbox tier (hereafter referred to as nestbox tier timing). Because the nestbox tier contains the nestboxes but also a balcony (figure 1*a*), we used several other lines of evidence to show that this probably reflects nesting behaviour. We believe the measure reflects nesting behaviour, as (i) the high density of hens in the nestbox tier between 02.00 and 08.00 (figure 2) probably prevents them from roosting on the balcony and (ii) the distribution of the interval time between consecutive nestbox tier timing has a narrow spread around 24 h that seems specific to the morning (95% of the values falling within 23.3–24.9 h, compared with 20.1–27.8 h for the behaviour computed over the following 6 h hours (08.00–14.00); illustrated in electronic supplementary material, figure S1). We hypothesize that more dominant, aggressive, proactive individuals, would lay earlier in the morning than subordinate, docile, reactive individuals.

#### 2.3.4. Sleeping tier

Previous literature suggested that hens are highly motivated [32] to roost at night on the highest area [33,34], but in commercial aviaries, not all hens can be on the highest tier simultaneously. On average across all available days, 69.4 ± 5.1% (mean ± s.d.) of the hens spent the night on the highest tier, 14.1 ± 4.7% on the nestbox tier, 16.2 ± 3.4% on the lower tier and 0.3 ± 0.7% on the littered floor. Therefore, we used a binary variable whether the hen spent most of the night-time on the highest tier (yes/no) as a behaviour related to the night-time routine which is hereafter referred to as sleeping tier. The night phase is preceded by 15 min dusk phase, where light is slowly reduced. We hypothesized that hens with a more proactive personality would have some traits (e.g. more bold, risk-taking, aggressive or faster and more active behavioural response [6] such as to the reduced light) that would enhance opportunities to roost at night on the highest tier.

#### 2.3.5. Feed delivery response

Feed is delivered automatically six times throughout the day (02.30, 06.00, 09.00, 12.00, 14.15, 16.15; figure 2) via an automatic chain feeding system that runs for three minutes at each delivery and produces an elevated noise level. Although hens had ad libitum access to feed, the feed delivery brings fresh particles of larger size that are generally preferred by chickens [35–37]. Hereafter, we will refer to the two tiers where feed is accessible (the lower and highest tiers) as the ‘feed-tiers’. We defined a feed delivery response as a descriptor of the tendency of being in a feed-tier more frequently while the fresh feed is delivered than while the feed is not delivered.

$$\mathrm{Feed}\;\mathrm{delivery}\;\mathrm{response}=\frac{1}{\left|P\right|}\underset{\;p\in P}{\sum }\left(\frac{1}{50}\overset{50}{\underset{r=0}{\sum }}\;\left(\frac{T_{p}\;\text{–}\;T_{\;pr}}{T_{p}\;+\;T_{\;pr}\;}\right)\right)\;,$$

where *P* corresponds to the set of periods when the feed is delivered, *T_p_* the time spent in the feed-tiers during the period *p*, and *T_pr_* the time spent in the feed-tiers during a random period (*pr*) of the same duration and around the same period of the day as *p*, but where feed was not delivered. Intuitively, the behaviour is similar to (*T_fd_* − *T_fnd_*)/(*T_fd_* + *T_fnd_*), where *T_fd_* (and *T_fnd_*) represent the daily time spent in the feed-tiers while fresh feed is delivered (and is not delivered), but where we controlled for the difference in duration between periods with feed delivery and without feed delivery. To do so, we compared each feed-delivery period (*p*) with 50 random periods without feed being delivered, but of the same duration and occuring within a 1 h window surrounding *p*, but disregarding the 15 minute period immediately before or after *p*. We believe 50 periods gave a reasonable representation of the overall behavioural patterns around the time of feed delivery (see electronic supplementary material, text S2 for further explanation). The feed delivery response varies from −1 to 1, where negative numbers indicate a tendency of being in the feed-tiers more frequently while the feed is not delivered, and positive numbers indicate a tendency of being in the feed-tiers more frequently while the feed is delivered. Because periods with reduced feeder space (as expected after arrival of fresh feed and suggested by figure 2) may be associated with increased aggression [38], we hypothesized that hens with higher values would generally be bolder as they risk greater aggression to get fresh feed compared with others, and thus we would deem them to be more proactive. To verify that this behaviour relates to the feed becoming available in the feed-tiers, we compared the behavioural responses computed with true and false feed delivery timing (defined as 20 min forward and 20 min backward in time). When computed with false timing, we found that on average 54 ± 5% or 53 ± 6% (forward or backward push, respectively) of individuals per day had a strictly positive response. When computed with true timing, we found substantially higher values, with on average 77 ± 4% of individuals per day having a strictly positive feed delivery response (illustrated in electronic supplementary material, figure S2b, d and f), indicating that the defined feed delivery response reflects hens' reaction to the feed being delivered.

### 2.4. Contexts

**Figure 3.**
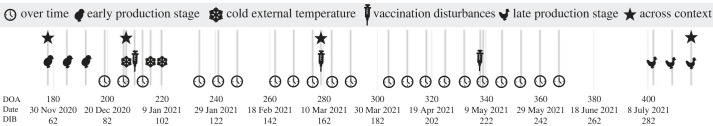
The timeline of the contexts in relation to the day of age (DOA), the date, and the number of days since the transfer to the laying barn (DIB). Each vertical bold grey line represents a day used in subsequent analysis. All days selected within a context, over time or across contexts are represented here.

To understand whether individuals varied their spatial behaviour across contexts, we selected two commonly occurring situations in commercial settings and two different production stages (for a total of four contexts). We chose an early and late production stage defined by the first and last three weeks of the tracking period to compare the extent of consistent individual differences from the onset of adulthood to the end of production, when animals are more likely to have poor welfare conditions. To include a common commercial perturbation, we used three vaccination events spread over 120 days, with each involving two hours of water deprivation (beginning at 08.00), followed by vaccination delivery through water, and a three-hour postponement of the WG opening. External environmental conditions, such as the temperature, may also influence behaviour, particularly when temperatures fall outside of the optimum thermoneutral zone, which in laying hens lies between 19°C and 22°C [39]. Therefore, we defined a ‘cold external temperature’ context, characterized by days with solely negative mean hourly external temperature during hours where access to the WG was provided (varying from −1°C to −4°C). Throughout the experiment, there were several periods characterized by consecutive days with negative temperatures. We chose the first day within each of the first three periods to limit habituation effects. Any day that would fit into more than one of the four contexts was excluded to avoid overlap between contexts. Days that fit into none of these contexts were considered to estimate repeatability over time but, due to possible autocorrelations between days close in time and uncontrolled human disturbances, we only used Saturdays (a day with limited human non-staff visits). Similarly, Saturdays were used for both the early and late production stage contexts. Thus, each context involved three distinct days, with at least 118 hens tracked during all 3 days. To evaluate the repeatability of behavioural differences across contexts, we used the observation in each of the four contexts that we believed was the most representative of the context, while avoiding observations close in time (detailed in figure 3). Subsequent analysis involved 194 hens and a total of 5047 observations, among which hens were tracked on average for 26 days (min = 1, max = 33, s.d. = 1, 25th percentile = 21, 50th percentile = 31, 75th percentile = 33 (unit: day)).

### 2.5. Statistical analysis

Analysis was conducted with Python for data processing and visualizations, and R for the statistics (code is given in the electronic supplementary material).

#### 2.5.1. Repeatability of behaviours

First, we evaluated individual consistency in each movement behaviour over time and across contexts. To evaluate individual consistency over time, we estimated the adjusted repeatability (defined as the proportion of the total variance that is accounted for by differences among individuals, while accounting for known individual differences), hereafter referred as repeatability, of each behaviour separately. Before addressing the across-contexts consistency, we investigated if individuals exhibited consistent behavioural differences within each context by estimating repeatability for each behaviour and context separately. Then, we computed the repeatability of behavioural differences across contexts. We used the rptR package [40] to calculate repeatability and fit a Gaussian distribution for both the vertical travelled distance behaviour and feed delivery response, and a binary distribution with logit-link function for both the WG presence and sleeping tier (i.e. went in the WG: yes/no; slept in one of the down tiers: yes/no), for which we reported the link-scale repeatabilities. We reported the repeatability estimate with its 95% confidence interval (CI) based on 1000 bootstraps. Because the nestbox tier timing is positively skewed, we used the glmmTMB package [41] with a gamma family and log-link function to extract the repeatability as explained in Stoffel *et al*. [42] (with the trigamma function to derive the observation-level variance) and used bootstrapping to get a mean estimate with confidence intervals, based on 1000 bootstrap replicates. We checked model assumptions (i.e. normality of error and homoscedasticity) by sight. Because consistent behavioural differences among individuals could arise from consistent external (environment) and internal (health) differences, also known as pseudo-repeatability [43], we accounted for KBF severity (which was already shown to be related to movement within the aviary [44] and is a prevalent health issue in commercial hens within aviaries [45,46]), body mass, class (MEXP/LEXP) and treatment (OFH/TRAN). Because there is a general upward trend over time for both the body mass and the KBF severity, we interpolated linearly (with monotonically increasing) both the KBF severity and body mass for each hen separately to better control for hens' health status between consecutive health assessments. To account for variation in the timing of feed delivery between pens due to sets of four pens being linked to a common feed delivery time, we controlled for a feed-chain identity in the feed delivery response. To facilitate model fit, we added a time effect (number of days since the transfer to the laying barn). All continuous fixed effects were scaled and centred to a mean of 0, so that intercepts reflect average values. The identity of the pen was previously tested for each behaviour separately and removed as the more complex models had greater Akaike information criterion (ΔAIC > 2) and a same conditional explained variance ($\Delta R_{\mathrm{conditional}}^{2}<0.01$). For model convergence issues, the class was removed for all models used to compute the repeatability of the WG presence ($\Delta R_{\mathrm{conditional}}^{2}=0$). In addition to the repeatabilities, we report the trait means as well as the within- and the among-individual variance components (see electronic supplementary material) [42].

#### 2.5.2. Behavioural syndromes

We evaluated behavioural syndromes, that is, correlations among individual's average behavioural expressions hereafter referred as ‘behavioural type’. We used the observations from all contexts in one multivariate model based on a Bayesian Markov chain Monte Carlo (MCMC) approach using the brms package [47] in R. We ran the model with uninformative priors, four Markov chains with 300 000 MCMC iterations (including 200 000 for burn-in), a thinning rate of 50, and with a similar distribution for the behaviour responses as in the models used to estimate the repeatabilities. To facilitate model fitting we scaled and centred (mean = 0, standard deviation = 1) the two Gaussian responses (vertical travelled distance and feed delivery response) and scaled the gamma response (nestbox tier timing) (standard deviation = 1). All continuous fixed effects were scaled and centred to a mean of 0 and standard deviation of 1. Specification of the model was assessed with posterior predictive check for each response variable, trace plots, Gelman–Rubin's convergence diagnostic [48], Geweke's convergence diagnostic [49] and leave-one-out cross-validation (Loo-cv). We reported the mean and credible interval of each among-individual correlation between any two behaviours. Correlations were deemed significant if the credible interval did not include zero [50] after rounding to three decimal places. To illustrate the use of these multivariate models to extract a behavioural axis that accounts for most of the among-individual variation in correlated behaviours, we performed an eigendecomposition of their among-individual correlation (so that all variables are given the same weighting even though measured on a different scale) matrix. We used the eigenvalues to report what proportion of variation is explained by each eigenvector (principal component) and reported the behavioural trait loadings on the eigenvectors with an eigenvalue greater than one [51]. We used the posterior samples to compute the standard deviation of each trait loading as uncertainty measure.

## 3. Results

### 3.1. Repeatability of behaviours

**Figure 4.**
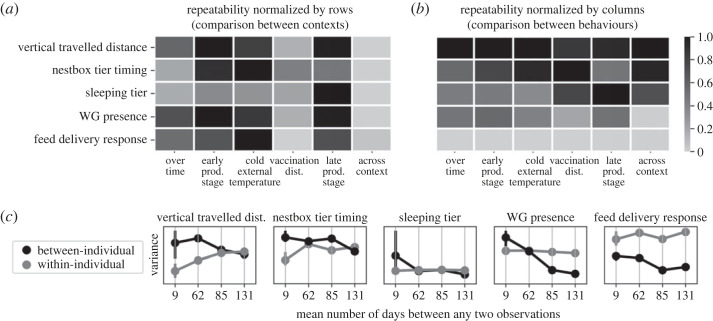
(*a*) Heat map of the adjusted repeatability estimates from table 1, where each row was normalized (between 0 and 1) to highlight the highest (black) and lowest (light grey) estimates for each behaviour and allow easy comparison between contexts. (*b*) Heat map of the adjusted repeatability estimates normalized by columns to highlight the highest (black) and lowest (light grey) estimates in each of the categories (over time, early and late life stage, cold external temperature, vaccination disturbances, and across context) and allow easy comparison between behaviours. (*c*) Illustration of the among- and within- individual variances underlying the repeatabilities, sorted by the ‘mean number of days between any two observations’ (from last row of table 1). This visual is intended to highlight the general trend of the individual variances when estimated on short or long interval time, the variances are further detailed in electronic supplementary material, table S1.

All behaviours were repeatable over time, within and across contexts, as none of our confidence intervals approached zero, indicating consistent inter-individual differences in our hen population. The most repeatable behaviour over time and across contexts was the vertical travelled distance (*R* = 0.66 [0.61, 0.70] and *R* = 0.48 [0.40, 0.56], respectively). The highest repeatability was for the sleeping tier within the late production stage context (*R* = 0.81 [0.65, 0.98]); the lowest repeatability was for the feed delivery response within the vaccination disturbances context (*R* = 0.23 [0.13, 0.35]). All estimates are provided in table 1 and a heat map of their normalized (row-wise and column-wise) estimates is shown in figure 4*a*. Normalizing each row, i.e. each behaviour separately, highlights for each behaviour which context is the most or least repeatable and therefore allows easy comparison of repeatability between context (e.g. if a context appeared as generally the least repeatable for all behaviours). Similarly, normalizing each column, i.e. each context separately, allows visualizing for each context which behaviour is the most or least repeatable, and thus allows easy comparison of repeatability scores between behaviours (figure 4*b*). Finally, the general trend of an association between the between- and within-individual variances with the mean number of days between any two observations (as provided by the last row of table 1) is provided in figure 4*c*, where we generally observe a lower between-individual variance and a higher within-individual variance over longer time periods. The trait means, the between- and the within-individual variance components are reported in electronic supplementary material, table S1.

**Table 1.** Adjusted repeatability of the five behaviours over time, within each context and across contexts (in the first five rows). In the following rows, we added information on the data used to compute the repeatabilities: the number of observations, individuals, days and hens with more than 95% of observations included (in the following three rows); the mean and maximum number of days between any two observations (in the last row).

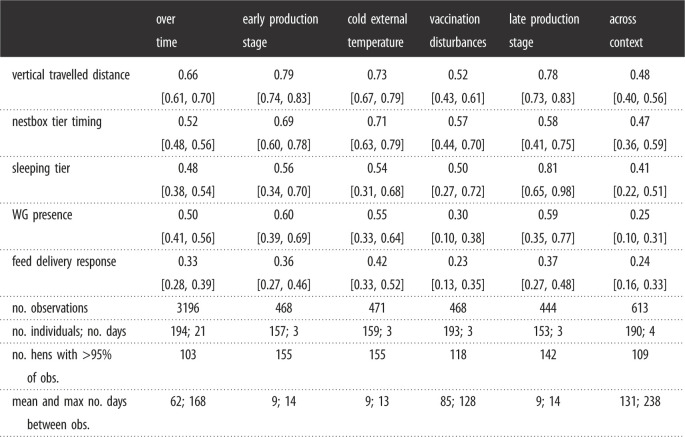

### 3.2. Behavioural syndromes

**Figure 5.**
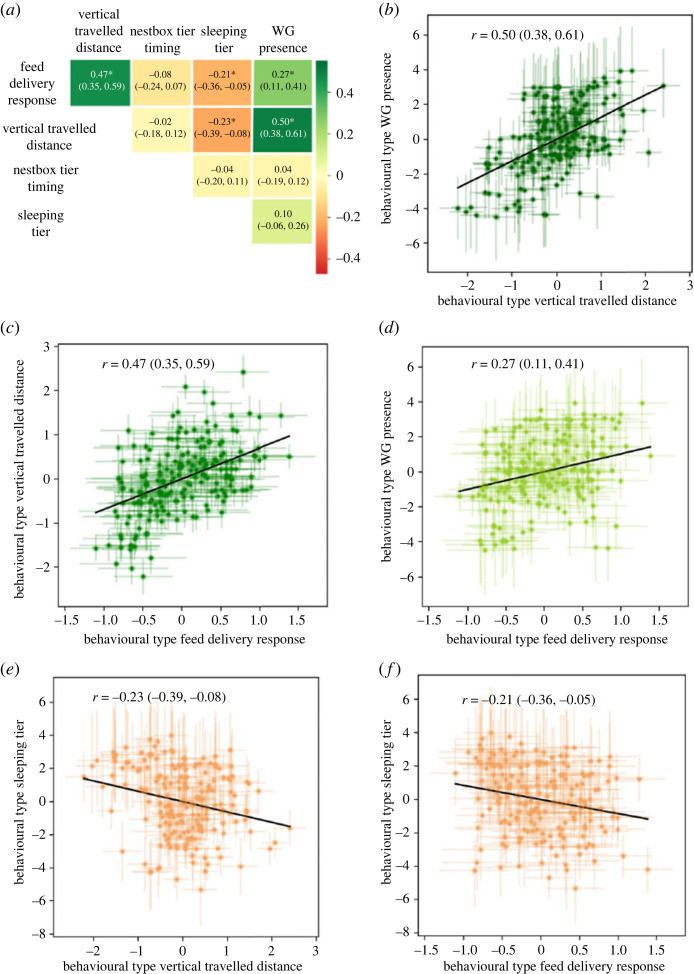
Correlations (±95% credible interval) of each movement behaviour from the multivariate mixed model where stars (*) highlight significant correlations (i.e. when 0 is not included in the confidence interval) in (*a*), illustrated by individual intercept estimates (±95% credible interval) of each correlated pair of behaviours: in (*b*) the WG presence and the vertical travelled distance, in (*c*) vertical travelled distance and the feed delivery response, in (*d*) the WG presence and the feed delivery response, in (*e*) sleeping tier and the vertical travelled distance, and in (*f*) sleeping tier and the feed delivery response. The slope from each regression line between these pairs of behaviours is represented by a black line and calculated by dividing the covariance between both behaviours with the variance of the behaviour displayed on the *x*-axis. Negative correlations are represented by red colours and positive correlations by green colours.

Results revealed existence of behavioural syndromes, involving four of the five behaviours. The vertical travelled distance was positively correlated with the WG presence (*r* = 0.50 [0.38, 0.61]) and feed delivery response (*r* = 0.47 [0.35, 0.59]), as well as negatively correlated with sleeping tier behaviour (*r* = −0.23 [−0.39, −0.08]). The feed delivery response and the WG presence were positively correlated (*r* = 0.27 [0.11, 0.41]). The feed delivery response and the sleeping tier were negatively correlated (*r* = −0.21 [−0.36, −0.05]). Correlations between each pair of behaviours and statistically significant correlations are illustrated in figure 5.

At the population level, the number of days spent in the barn had the largest effect on all five behaviours. With increasing days in the barn, hens on average visited the WG on more days, spent fewer nights on the highest tier, reduced their vertical travelled distance per hour spent indoors, used the nestbox tier later (as measured by a greater response of the nestbox tier timing behaviour) and increased their tendency to be in the feed-tiers upon feed delivery feed (the observed behaviours over time are illustrated in electronic supplementary material, figure S3). Also, heavier birds used the nestbox tier earlier and hens that travelled greater vertical distances had more severe keel bone fracture scores and greater mass. Treatment (OFH/TRAN) had no effect. The more/less explorer class effect was statistically significant for only one behaviour where more explorative hens were found to spend fewer nights on the highest tier compared with hens randomly selected among the entire population. Estimates of population-level effects are further detailed in electronic supplementary material, figure S4.

**Figure 6.**
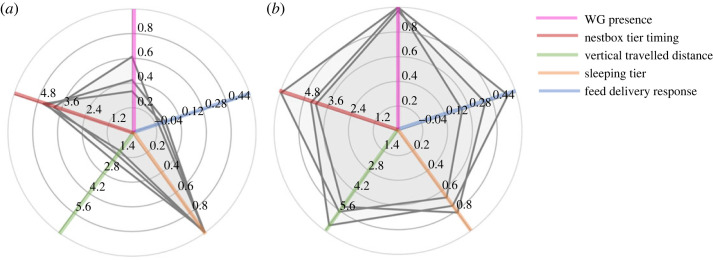
Illustration of two ‘behavioural profiles’ as suggested by the first eigenvector of the among-individual correlation matrix. We randomly selected three hens among the 15% lowest (*a*) and highest (*b*) mean score to visualize their mean observed behaviours, including the nestbox tier timing behaviour, shown to be independent of the other behaviours.

The eigendecomposition of the among-individual correlation matrix based on the correlated behaviours (all behaviours except nestbox tier timing) resulted in two principal components with an eigenvalue greater than 1, and explained 47% and 28% of the total variation, respectively. On the first principal component, the sleeping tier weakly loaded in the opposite direction to the feed delivery response, the WG presence and the vertical travelled distance. Behavioural traits loadings (mean ± s.d.) on the first and second eigenvectors of the among-individual correlation matrix were: feed delivery response: 0.54 ± 0.03 and −0.16 ± 0.09, vertical travelled distance: 0.63 ± 0.02 and 0.03 ± 0.05, sleeping tier: −0.24 ± 0.09 and 0.83 ± 0.07, WG presence: 0.50 ± 0.04 and 0.54 ± 0.11, respectively. The first principal component suggests a behavioural axis that accounts for most of the among-individual variation in correlated behaviours. Although individuals varied along a continuum on the axis, the extremes can illustrate different ‘behavioural profiles' within the flock. To illustrate these extremes, we projected observed behaviours of each selected day onto the subspace spanned by the first component and randomly selected three hens among the 15% highest and lowest mean score to visualize their mean observed behaviours (figure 6).

## 4. Discussion

This is the first study to quantify long-term individual consistency in various spatial behaviours of commercial hens within an aviary system. The behaviours were selected to represent five aspects of commercial hens' daily routine, and therefore may be functionally different. We found consistent individual differences over time and across contexts in the five behaviours. The daily vertical travelled distance and the nestbox tier timing behaviours were most repeatable. We also found behavioural syndromes involving the sleeping, feeding, indoor movements and outdoor usage behaviours, but interestingly not the nesting-related behaviour, suggesting two behavioural axes driven by different mechanisms. Altogether, these long-term individual consistencies and behavioural correlations revealed the potential applicability of such behaviours as personality traits in commercial hens and suggest two main axes of spatial personalities.

### 4.1. Repeatability of behaviours

Consistent inter-individual differences in behaviour may arise from intrinsic individual (genetic or epigenetic) differences, but also from consistent external and internal differences (e.g. bone fractures) between individuals. In commercial settings, numerous sources of variation are under strict human control (e.g. rearing process, light and indoor temperature) which may reduce consistent external differences between individuals and in turn avoid spuriously high repeatabilities. In addition to offering unique conditions for controlling and standardizing environmental conditions, commercial settings often have automated processes (e.g. to provide feed or access to areas) that could be manipulated and incorporated into the design of experiments. We highlight that animals with known genetics, standardized routines and housed in commercial settings offer an excellent system to study behavioural variation at different hierarchical levels.

For the design of future experiments, it is important to understand the effect of interval time between observations on the repeatability of behaviour. Our repeatability estimates decreased with increasing mean interval time between observations, as expected [52]. The underlying individual variances suggest that hens have more variable behaviours (i.e. higher within-individual variance) and are behaviourally more alike (i.e. lower between-individual variance) when studied over longer periods. The observed trends from figure 4*c* highlight the two underlying mechanisms by which repeatability of behaviours generally reduced over longer observational periods, though the factors driving the mechanisms remain unknown and probably include both internal and external changes.

Repeatability may also vary with respect to an individual's life stage (e.g. due to ontogeny [53]), yet we found similar repeatabilities during our early and late production stages. However, we previously found that similar behaviours expressed previous to our early production stage on the same hens (during the first two months in the laying barn) were considerably less repeatable (*R* varying from 0.38 for the vertical travelled distance to 0.17 for the sleeping tier) [54]. These results show that hens were already behaviourally consistent prior to our early production stage, but that repeatability increased and stabilized during the first two months in the laying barn and seemed to be maintained until the end of production.

We found consistent individual differences in all behaviours over time and across contexts, revealing their potential applicability as personality traits of commercial laying hens. Especially the daily vertical travelled distance and the nestbox tier timing, for which consistent differences between individuals explained more than half of their variation (66% and 52%, respectively) and were maintained across contexts (48% and 47%, respectively). By contrast to these moderately high long-term repeatabilities, consistent differences in both the WG presence and the feed delivery response were only weakly maintained across contexts (*R* = 0.25 and *R* = 0.24, respectively), suggesting these behaviours may be of lesser importance to the hens or harder to maintain (e.g. due to behavioural plasticity in response to the outside temperature; or during the vaccination disturbances context). Also, because not all hens can be on the feed-tiers simultaneously due to the limited space, it may limit hens to express consistent feed delivery responses, which could explain the general low repeatabilities of the behaviour.

Although the feed delivery response did not show drastic behavioural differences within our population, it may be of particular relevance in commercial settings where animals have to regularly respond to external stimuli. It is important to note that as we are unable to control for all internal or external drivers, this behaviour may not directly reflect animals' motivation to feed. However, it is a first step towards assessing a potential proxy of the animal's affective state in response to a recurring external stimulus based on movement data. Further research should assess how such behaviours are indicative of animal well-being, for example by testing if hens with higher feed delivery responses also have a more optimistic attitude in a judgement bias test [55]. If supported, the behaviour could then be used as a daily proxy of the hens’ affective state.

Altogether, these long-term repeatabilities demonstrated that tracking technology can be used to quantify individual differences in behaviours related to different aspects of commercial hens' routine and for long periods that are rarely accounted for in personality studies. These results demonstrated that hens from a single parent flock can differ consistently in spatial behaviours over most of the production period, suggesting that individuals have different preferences or needs. Such individual preferences may lead to overcrowding or under-used areas, which could affect animal welfare (e.g. smothered hens in overcrowded areas [56]) and production (e.g. eggs on the floor due to preferred nestbox being occupied [57]). Therefore, by understanding behavioural differences in commercial settings we can design appropriate management tools and breeding practices to improve animal welfare, such as sensors to detect pilling behaviours [56], practices to encourage earlier transitions between tiers after transfer to an aviary [58], and breeding hens for specific behaviour [17,57].

### 4.2. Behavioural syndrome

In accordance with our predictions, we found that hens that travelled greater vertical distances, on average also went in the WG on more days and tended to use the feed-tiers more upon delivery of fresh feed. These results support our hypothesis that these behaviours are associated with a pro-reactive personality axis. We proposed that these behavioural expressions are indicative of a proactive personality. Contrary to our predictions, these hens on average also use the highest tier slightly less at night (correlation with vertical travelled distance: *r* = −0.23). Because hens are highly motivated [32] to roost on the highest tier [33,34], this result could suggest that more proactive hens are less successful in accessing the highest tier at night. For instance, hens that travel greater vertical distances may also stay active until later and, therefore, be less able to access the highest tier due to higher animal densities. Further research is required to evaluate associations between these behaviours and common personality traits to understand the role of pro-reactive personality axis in explaining these syndromes.

An alternative, non-mutually exclusive, mechanism to explain these behavioural differences and syndromes could be the existence of subgroups, where hens’ location would reflect those of their group. In large groups such as ours, hens cannot recognize all conspecifics, which probably limits their ability to form social groups based on individual recognition [59]. Therefore, we believe it is unlikely that hens repeatedly associate their locations with the same individuals due to such social groups. However, hens may recognize conspecific or relevant traits (e.g. comb size) based on their status [60]. Therefore, subgroups could reflect dominance rank, and hens' location would reflect their rank. Future research should investigate the social dynamics of these large groups and whether this could explain spatial behavioural differences.

Interestingly, we found no syndrome involving the nesting behaviour, which suggests that its main mechanism is independent of those involved in the other behaviours and could be, for instance, the physiological rhythm. Because commercial laying hens are under strong human selection for high egg production, we may speculate that the behaviour is more resilient to environmental change and could be expressed independently to other needs and their associated behaviours. Altogether, these correlations indicate two main axes of spatial personalities: the nesting-related behaviour and the behavioural axis based on the other behaviours. We used the extreme values from the latter axis to illustrate two, probably most dissimilar, behavioural profiles in our flock (figure 6). This figure illustrates how this one axis is accounting for most of the among-individual variation and therefore may be used to extract relevant phenotypes.

To assess relative benefits of these phenotypes, future research should evaluate not only their associations with common personality traits but also with animal welfare and productivity. According to Koolhaas [61], proactive animals perform better under highly predictable conditions, or when feed is abundant [62], compared with reactive animals. However, in commercial settings, animals often have a predictable management daily routine, but unpredictable events such as diseases and vaccinations frequently occur. Therefore, it is unclear which of the proactive or reactive personalities would perform better in such settings. A greater understanding of how personality traits relate to welfare and production could inform on potential phenotypes to integrate into the breeding process for more robust farm animals [4,14,15,17]. Our repeatability estimates set a promising upper bound on the heritability of these behaviours and the behavioural syndromes highlight some potential constraints for selection. In conclusion, this study supports the use of tracking technology to assess behavioural traits and potentially breed more robust farm animals.

## Data Availability

The data and code for this study are available on https://doi.org/10.17605/OSF.IO/63PQ5 [63]. The data are provided in electronic supplementary material [64].
